# The clinical utility of contrast enhanced whole-heart coronary MRA with 32-channel coil at 3T scanner in the era of 64 and more-slice CT

**DOI:** 10.1186/1532-429X-15-S1-W28

**Published:** 2013-01-30

**Authors:** M Wu, Y Huang, F Wu, K Chiou, H Huang

**Affiliations:** 1 Radiology, Kaohsiung Veterans General Hospital, Kaohsiung, Taiwan; 2 Radiology, Faculty of Medicine, School of Medicine, National Yang Ming University, Taipei, Taiwan; 3 Cardiology, Kaohsiung Veterans General Hospital, Kaohsiung, Taiwan

## Background

Sixty-four and more-slice cardiac CT provides rapid and high quality coronary CT angiography for the majority of clinical needs of non-invasive evaluation of coronary artery. However, the recent advance of whole-heart coronary MRA has been able to provide high-quality of coronary MRA and also myocardial image in a reasonable scan time. The purpose of this study was to evaluate the clinical indication and the feasibility of coronary MRA with 32-channel coil at 3T scanner.

## Methods

Acquisition of 3.0T coronary MRA data was done by using 32-channel torso coil (Skyra, Siemens AG Healthcare, Erlangen, Germany). An ECG-triggered, respiratory navigator-gated, inversion-recovery prepared, segmented gradient-echo sequence was used for image acquisition with an acceleration factor of 3 in the phase-encoding direction using generalized auto calibrating partially parallel acquisitions reconstruction. Slow infusion of 0.15 mmol/kg body weight of Gadobenate dimeglumine (MultiHance; Bracco Imaging SpA, Milan, Italy) at a rate of 0.2 mL/s was given. The image quality of coronary MRA was scored as 4, excellent; 3, good, confident for diagnosis; 2. fair, suboptimal for diagnosis; and 1, poor, not diagnostic. The clinical impact of the examination were classified as high, major revision; medium, minor revision or confirmation and low, non contribution to clinical diagnosis and management.

## Results

Totally 76 patients (median of age = 22 years, 55 male) received the examination and 71 completed. The 5 failures were due to interrupted anesthesia (N =3), unstable vital signs (N=2). The indications of the 71 examination were (1). Kawasaki disease, N= 26 ; (2). anomalous coronary artery, with or without complex congenital heart disease, N = 8; (3). assessment of coronary artery disease in young patients with familiar hyperlipidemia, N = 9 ; (4). assessment of coronary artery stenosis in patients with heavy calcification, N = 11 and (5). complete evaluation of newly onset heart failure with low risk of coronary artery disease, N = 17. The scores of image quality were 18, 42, 8, 3 for scores of 4, 3, 2, and 1, respectively. The mean image time was 8.2 min. There were additional image findings included: 1. hyper-enhancement of myocardium, N = 23; 2. myocardial wall hypertrophy, N =17; 3. myocardial wall focal thinning, N = 14; 4. functional valvular disorders, N = 7. The clinical impacts of the examination were high in 14, medium in 40 and low in 17 patients.

**Figure 1:**
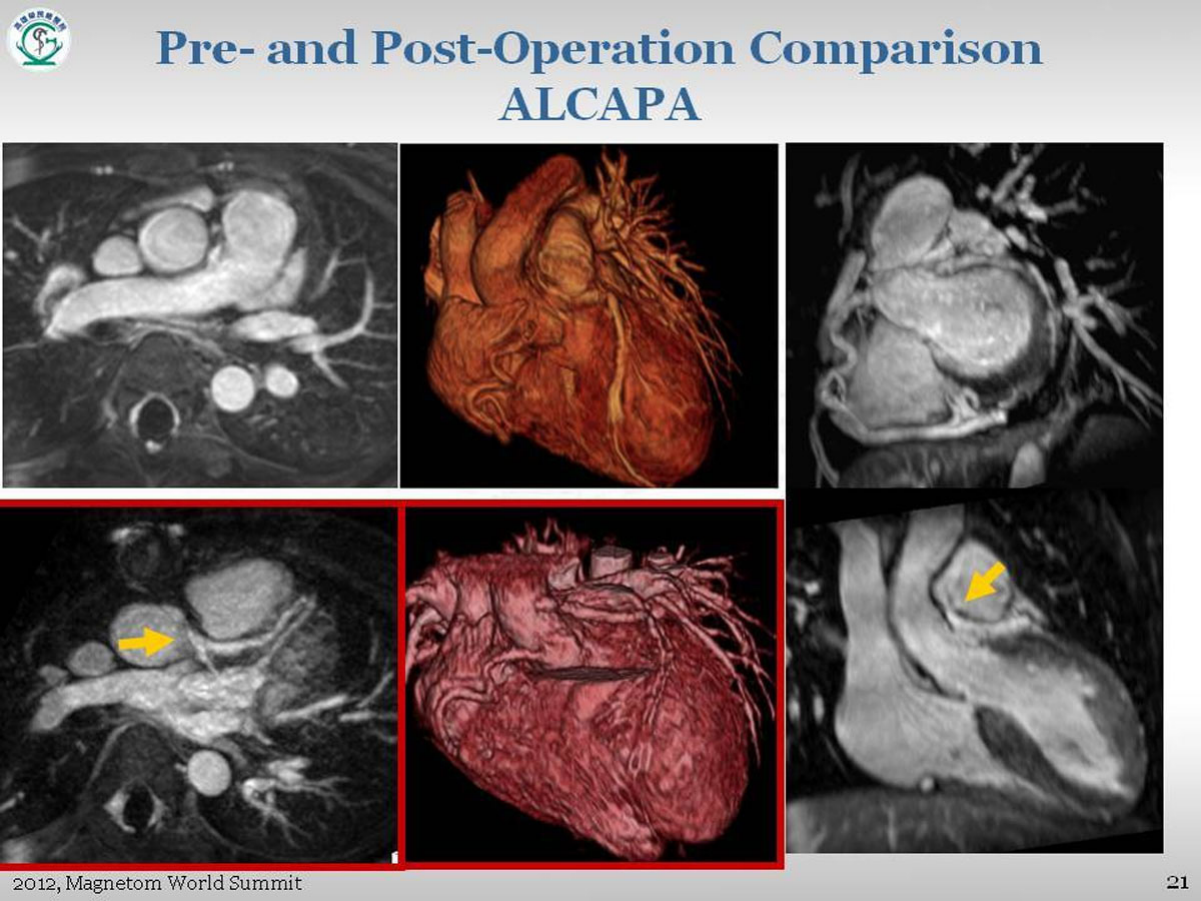
Pre-operation: Aabnormal origin of left coronary artery from the inferior wall of main pulmonary artery, which has patent anastomosis with right coronary artery. Postoperation (lower-right): In-plantation of left coronary artery to the aorta

## Conclusions

Contrast enhanced whole-heart coronary MRA with 32-channel coil at 3T scanner has high clinical feasibility for appropriate clinical indications.

